# Frictional Characteristics and Tribological Mechanisms of Ionic Liquid Lubricants in Ceramic Tribo-Systems

**DOI:** 10.3390/ma18194504

**Published:** 2025-09-27

**Authors:** Zehui Yang, Shujuan Li, Limu Cui, Congjun Cao

**Affiliations:** School of Mechanical and Precision Instrument Engineering, Xi’an University of Technology, Xi’an 710048, China

**Keywords:** tribochemistry, ionic liquid, lubricants, ceramics, tribochemical reaction

## Abstract

Due to their excellent mechanical stability, chemical stability, and environmentally friendly properties, ceramic materials have received extensive attention for years. Meanwhile, ionic liquids (ILs) have been found to effectively enhance tribological properties when applied as lubricants, which has become a distinctive example of their wide exploration. Here, three novel proton-type ionic liquids containing different polar groups were designed and synthesized as pure lubricants for use on different ceramic friction couples (silicon nitride–silicon nitride, silicon nitride–silicon carbide, and silicon nitride–zirconium oxide contacts), and their lubrication effect was evident. The results indicate that the adsorption behavior and frictional characteristics of different polar groups on a ceramic friction interface differ, largely depending on tribochemical reactions and the formation of a double electric layer on the interface between the ILs and ceramic substrates, without obvious corrosion during sliding. The friction coefficient is reduced by more than 80%, and this excellent anti-friction effect demonstrates that the constructed ionic liquid–ceramic interface tribological system shows good application potential. Based on the analyses of SEM, EDS, and XPS, the tribochemical reaction on the sliding asperity and the film-forming effect were identified as the dominant lubrication mechanisms. Here, the high lubricity and anti-wear performance of ILs containing phosphorus elements on different ceramic contacts is emphasized, enriching the promising application of high-performance ILs for macroscale, high-efficiency lubrication and low wear, which is of significance for engineering and practical applications.

## 1. Introduction

Ceramic is an inorganic nonmetallic compound rather than a simple substance, whose crystal structure is complex and diverse. The bonding between elements for ceramic is mainly ionic and covalent [1,2,3]. In practical application, the chemical bond of most ceramics is a mixture between an ionic bond and covalent bond [4]. Therefore, compared with ordinary metals, the structure for ceramic is complex and their surface energy is high, so their strength, elastic modulus, wear resistance, and corrosion resistance are superior to those of metallic materials [5]. However, in terms of plasticity, machinability, and seismic resistance, the performance is relatively poor. Therefore, their applications in some cases are limited. In addition, owing to their advantages, ceramics have replaced metallic materials in some fields [3,6].

High-performance structural ceramics are widely used in mechanical engineering [7]. On the one hand, this is due to the mature preparation technology of engineering ceramics; on the other hand, it is due to their unique properties, including the high hardness and strength, high compressive strength, low tensile strength, low density, good chemical inertia, excellent corrosion resistance, and anti-wear performance [8]. Interestingly, engineering ceramics do not show the shortcomings (easy oxidation and corrosion) of traditional metallic materials [9,10]. In addition, these special properties give engineering ceramics excellent high-temperature resistance, oxidation resistance, good high-temperature strength, and wear and corrosion resistance, making them suitable for various application fields, such as the automobile industry (cylinder liner, valve seat and guide, piston crown, and turbocharger parts), petrochemicals, high-temperature cutting tools, aerospace, the building materials and mineral industry, etc. Among them, high temperature, anti-corrosion, wear-resistant materials play an especially important role. At present, there are many kinds of engineering ceramics. Among them, silicon nitride (SIC), silicon nitride (Si_3_N_4_), and zirconia (ZrO_2_) are representative ceramic materials, which are characterized by a high structural strength and high thermal conductivity [11] and a low thermal expansion coefficient. It is applicable for use in high-temperature service environments, mainly in rocket engine nozzles, cutting tools, thermocouple protection tubes, furnace linings, high-temperature rotating ball-bearing parts, etc. [12].

Sliding friction, a high sliding speed, severe wear, and other lubrication problems have a negative impact on the meshing points, contact area, and moving parts of mechanical equipment. Therefore, high-performance lubricants will be innovated and developed rapidly [13,14,15], providing stable lubrication under extreme working conditions (high temperature, high pressure, and strong corrosion), solving the problem of traditional lubricating oil/grease failure and ensuring the reliable operation of equipment, as well as extending its service life [16,17]. At present, solid lubricants, polyols, high-temperature greases, water-based lubricants, and silicone oil/fluorine oil show excellent lubricity and service stability for ceramics. In the shear process, they react with the matrix through strong interactions, leading to the formation of a boundary protective layer and a reduction in severe wear. However, these lubricants have some disadvantages. For example, the application of water-based lubricants is limited by test temperatures due to their high volatility, low viscosity, poor film-forming ability, and high freezing point [18]. In addition, the tribochemical reaction film formed on the contact substrate is very thin and unstable under the lubricating effect of aqueous lubricants, resulting in a high friction coefficient and severe contact wear. Polyols are also not ideal lubricants for the inherent limitation of their low boiling point and high vapor pressure. Therefore, aqueous lubricating materials and polyols are not suitable for the stable and efficient lubrication of the SiC, Si_3_N_4_, and ZrO_2_ ceramics interface in a harsh environment [19,20].

Ionic liquids (ILs), also known as room-temperature molten salt, are organic liquid substances composed of organic or inorganic cations and anions that remain in liquid form at or near room temperature [9,21,22]. They possess a number of unique advantages—such as a low vapor pressure, high ionic conductivity, high solubility of metal salts, and strong polarity—that have enabled their wide use in organic synthesis, catalysis, separation, colloidal interface, and nanomaterials. ILs were first reported as a new excellent lubricant by Ye et al., 2001 [23]. Later, more and more attention has been paid to ILs as a lubricant, and they have been widely studied by many researchers [24,25,26,27,28,29,30]. Previous studies have demonstrated that ILs exhibit remarkable anti-wear and friction-reducing properties, functioning both as neat lubricants and as lubricant additives. The molecular structure and cation–anion pairing of ILs are recognized as key factors governing their tribological performance [29,31,32,33,34,35].

More than one million anions and cationic combinations have already been proposed for ILs, offering broad application potential and diverse tribological behaviors. In addition, the previous results show that the traditional anti-wear lubricants containing phosphorus and sulfur are known to provide superior lubrication, which highlights the importance of incorporating such polar elements into IL design. For ceramic substrates, however, the self-assembly behavior and tribological properties of ionic liquids vary significantly with their molecular structures. Investigating the structure–activity relationship between molecular structures and lubrication performance is, therefore, essential for developing high-performance IL–ceramic tribological systems. To this end, novel ionic liquid lubricants (containing the sulfur, nitrogen, and phosphorus polar group) were designed, and their physico-chemical properties and tribological performance were systematically evaluated on various ceramic contacts (Si_3_N_4_–SiC, Si_3_N_4_–Si_3_N_4_, and Si_3_N_4_–ZrO_2_ contact). Furthermore, the interactions among molecular structure, substrate characteristics, and lubrication behavior were analyzed. X-ray photoelectron spectroscopy (XPS) was employed to elucidate the underlying lubrication mechanisms. This work provides valuable theoretical guidance for the rational design and performance optimization of advanced lubricants tailored for ceramic applications.

## 2. Experiment Details

### 2.1. Materials

The materials used were sulfuric acid (98%, Xilong Chemical Co., Ltd., Shenyang, China), nitric acid (65%~68%, Xilong Chemical Co., Ltd., Shenyang, China), phosphoric acid (95%, Xilong Chemical Co., Ltd., Shenyang, China), and N, N-Dimethylformamide (DMF, 99%, Xilong Chemical Co., Ltd., Shenyang, China). All reagents were of analytical grade and were used directly without further purification.

### 2.2. Preparation of IL

**Figure 1:**
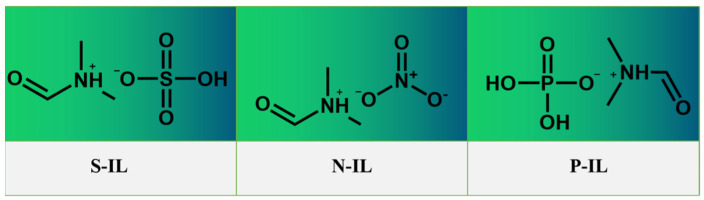
Molecular structure formula of ionic liquid lubricants.

N,N-Dimethylformamide was placed in a round-bottomed flask and cooled in an ice bath. Under continuous stirring, acids with different polar groups were slowly added to the flask via a constant-pressure dropping funnel. The mixture was then stirred at 80 °C under a nitrogen atmosphere for 12 h. The resulting PILs were dried under high vacuum for 24 h to minimize residual moisture. The molecular structures of the synthesized IL lubricants are shown in Figure 1.

### 2.3. Characterizations and Instruments

The structures of the synthesized IL lubricants were characterized by nuclear magnetic resonance (NMR) spectroscopy using an Agilent 400 MHz spectrometer (Santa Clara, CA, USA). Fourier-transform infrared spectroscopy (FTIR, Frontier, PerkinElmer, Singapore) was employed to identify the functional groups of the ILs, with a wavenumber range of 400–4000 cm^−1^. Thermogravimetric analysis (TG, STA 449F3, NETZSCH, Selb, Germany) was performed under nitrogen to determine the thermal decomposition temperatures of the synthetic ester oils (heating range: 30–600 °C, rate: 10 °C per minute). The viscosities of the IL lubricants were measured using a kinematic viscosity tester at 25 °C. To evaluate the corrosion resistance of different-molecular-structure ILs on ceramic substrates, corrosion tests were conducted for the test conditions that the temperature of 100 °C and the test time of 24 h. Polished SiC, Si_3_N_4_, and ZrO_2_ ceramic blocks (10 mm × 10 mm) were used as substrates, onto which 0.2 mL of each IL was dropped. After the test, the ILs were removed, and the ceramic plates were cleaned with ethanol. The surface morphologies of the SiC, Si_3_N_4_, and ZrO_2_ ceramics were then examined using scanning electron microscopy (SEM, FEI Quanta FEG 250, Portland, OR, USA), accompanied by energy-dispersive X-ray spectroscopy (EDS, EDAX Genesis APEX APOLLO X, 20 kV, Beijing, China) for elemental mapping of the protective layers on the ceramic substrates.

### 2.4. Tribological Performance

The tribological behaviors (anti-friction and anti-wear properties) of the IL lubricants on SiC, Si_3_N_4_, and ZrO_2_ ceramic contacts were evaluated using a conventional pin-on-disc reciprocating tribometer (Rtec, San Jose, CA, USA). The tests were performed with Si_3_N_4_ ceramic balls (diameter: 6.35 mm) as pins and freshly polished SiC, Si_3_N_4_, and ZrO_2_ ceramic blocks (20 mm × 20 mm) as discs. Test conditions included normal loads of 5 N, 10 N, and 20 N, a frequency of 1 Hz, an amplitude of 5 mm, and a relative humidity of 25–35%. All ceramic balls and discs were ultrasonically cleaned prior to testing. For each tribological test, 0.2 mL of ionic liquid lubricant was applied. The wear of the ceramic discs after friction tests were detected by MicroXAM 3D noncontact surface mapping microscope profiler (BRUKER-NPFLEX, Billerica, MA, USA). The lubrication mechanisms of P-IL lubricants on SiC, Si_3_N_4_, and ZrO_2_ ceramics were further investigated by analyzing the distribution of element valence states on worn surfaces using X-ray photoelectron spectroscopy (XPS, PHI 5000 VersaProbe III, Tokyo, Japan).

## 3. Results and Discussion

### 3.1. Physicochemical Properties

#### 3.1.1. Structure Characterization

The structures and purities of IL lubricants were finely confirmed by ^1^H NMR and ^13^C NMR. These proton-based ionic liquid are straightforward to synthesize and do not require a complicated purification process, thereby providing a practical basis for potential industrial applications.

**S-IL:** ^1^H NMR (400 MHz, DMSO)**δ:** 2.85–2.87 (t, 6H), 4.05 (s, 1H), 7.36 (s, 1H), 8.8 (s, 1H). ^13^C NMR (100 MHz, DMSO)**δ:** 40.2, 185.6.

**N-IL:** ^1^H NMR (400 MHz, DMSO)**δ:** 2.86–2.88 (t, 6H), 4.75 (s, 2H), 7.32 (s, 1H), 9.0 (s, 1H). ^13^C NMR (100 MHz, DMSO)**δ:** 37.4, 188.9.

**P-IL:** ^1^H NMR (400 MHz, DMSO)**δ:** 2.88–2.90 (t, 6H), 4.66 (s, 2H), 7.22 (s, 1H), 8.9 (s, 1H). ^13^C NMR (100 MHz, DMSO)**δ:** 38.6, 184.9.

#### 3.1.2. Thermogravimetric Analysis

**Figure 2:**
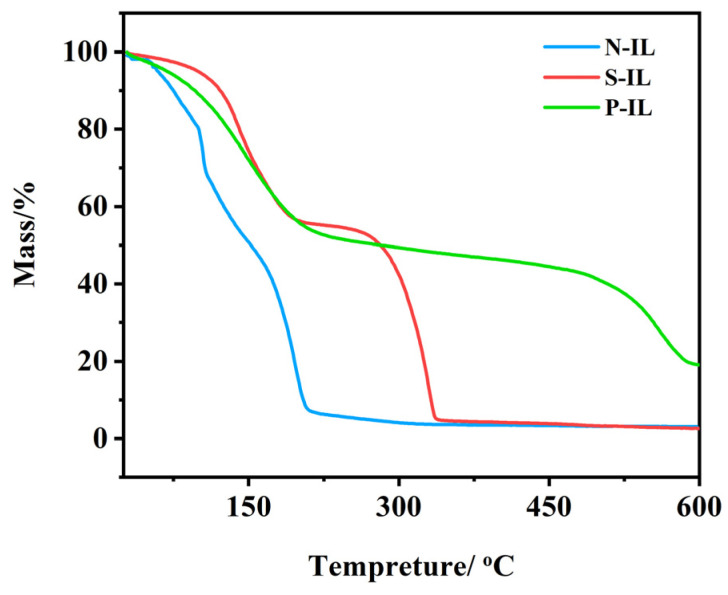
Thermal decomposition temperature curve of ionic liquid lubricants.

The excellent thermal stability of ionic liquids enables them to remain stable under high-temperature conditions, which is closely related to their molecular structure and ionic interactions. As shown in Figure 2, the thermogravimetric curve reveals that N-IL decomposes first, likely due to the instability of the anions in its ionic liquid structure [36]. At elevated temperatures, the C–N bond within the cation is prone to homolytic or heterolytic cleavage. For instance, quaternary ammonium cations may undergo Hoffman elimination reactions, yielding olefins and amine structures. In general, the stability of ionic liquids arises from the electrostatic attraction between cations and anions, which facilitates the formation of stable molecular aggregates. High temperatures disrupt this electrostatic interaction, enhancing the relative mobility of the ions and thereby accelerating cation decomposition. This leads to a reduction in the overall thermal stability. It can, thus, be inferred that, under combined thermal and mechanical stresses, ionic liquids undergo decomposition accompanied by complex chemical reactions. The thermogravimetric curves of S-IL and P-IL exhibit two distinct stages, indicating the generation of multiple thermal decomposition products under heat treatment.

#### 3.1.3. Viscosity

Viscosity is a critical property of lubricants, reflecting their internal resistance to shear flow [7]. If the viscosity is too low, the lubricant film becomes thin during sliding, creeps rapidly, and fails to adsorb effectively at the interface, leading to excessive friction. Conversely, if the viscosity is too high, excessive internal resistance arises, causing unstable lubrication. Therefore, a moderate viscosity is essential for favorable tribological behavior at the interface [37]. The viscosities of the three ionic liquids were measured at room temperature. S-IL (7.98 mm^2^/s) and N-IL (2.43 mm^2^/s) exhibited relatively low viscosities, whereas P-IL (95.75 mm^2^/s) showed a more suitable viscosity. Structurally, the type of anion exerts a dominant influence on viscosity; P-IL, with its bulky anion and high charge density, exhibited a substantially higher viscosity. This increased viscosity enhanced the load-bearing capacity of the lubricant film, providing the basis for its superior friction-reducing and anti-wear performance (Table 1).

### 3.2. Corrosion Analysis

**Figure 3:**
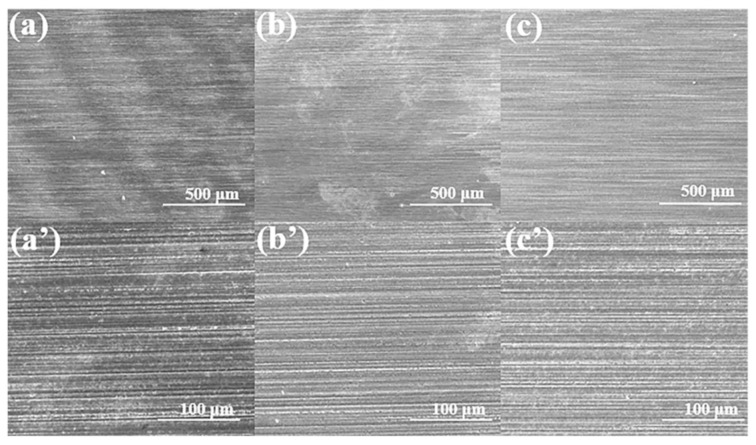
SEM micrographs after corrosion test on the Si_3_N_4_ ceramic interface by N-IL (**a**,**a′**), S-IL (**b**,**b′**), and P-IL (**c**,**c′**) lubricants.

**Figure 4:**
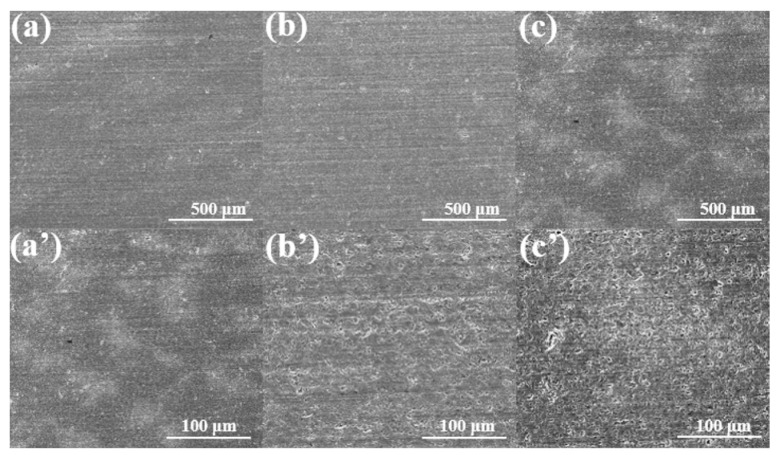
SEM micrographs after corrosion test on the SiC ceramic interface by N-IL (**a**,**a′**), S-IL (**b**,**b′**), and P-IL (**c**,**c′**) lubricants.

**Figure 5:**
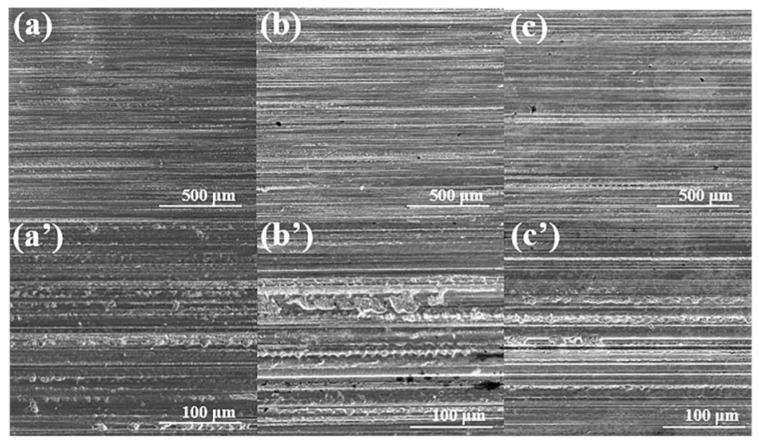
SEM micrographs after corrosion test on the ZrO_2_ ceramic interface by N-IL (**a**,**a′**), S-IL (**b**,**b′**), and P-IL (**c**,**c′**) lubricants.

The corrosion behavior of ILs on ceramic interfaces was evaluated by the bubble sheet test. The results reveal distinct corrosion behaviors on different ceramic surfaces, which depend not only on the composition of the ionic liquid and the water content within the molecules, but also on the intrinsic reactivity of the ceramic substrate [38]. Among the tested ceramics, Si_3_N_4_ exhibited the highest surface activity and superior interfacial properties. Here, high activity refers to the enhanced interfacial reactivity with metals or other materials at elevated temperatures, accompanied by a strong binding capacity. The localized temperature rise at the real-time frictional contact facilitates reactions between the ceramic substrate and highly polar ionic liquids. Consequently, tribochemical reactions occur at the interface, forming boundary protective films under combined mechanical and thermal stresses. The surface roughness showed no significant changes, indicating favorable corrosion resistance, as shown in Figure 3. In contrast, SiC displayed moderate activity (Figure 4). Pronounced pitting corrosion was observed after lubrication with N-IL. This phenomenon can be attributed to the thermal decomposition of N-IL, which generates corrosive products that further attack the substrate. Nitrate, as a strong oxidizing anion, readily releases highly corrosive oxides, particularly in the presence of metal catalysts. For the ZrO_2_ surface, corrosion was the most serious, with extensive surface corrosion products observed (Figure 5). However, the corrosion on the surface of P-IL is the least, and the corrosion on S-IL is the largest. These differences may be ascribed to the interactions between different polar anionic groups and ceramic substrates. SEM observations confirmed that phosphate-based ILs did not produce evident corrosion or excessive surface deposits, highlighting their superior protective performance. This difference in corrosivity arises primarily from the molecular structures of the ILs and their interactions with ceramic substrates. Structurally, N-IL is prone to decomposition, generating acidic products that aggressively corrode ceramic surfaces. The strong interaction between S-IL and the ceramic substrate, attributed to sulfur-containing groups, promotes severe corrosion. By contrast, the high viscosity and favorable chemical reactivity of P-IL enable the formation of a protective surface layer. The intrinsic stability of phosphate ILs prevents hydrolysis during testing, resulting in negligible surface corrosion. Moreover, the polar groups in the IL molecular structures can adsorb efficiently onto the porous ceramic surfaces, forming molecular adsorption layers that contribute to superior corrosion resistance.

### 3.3. Tribological Behavior

#### 3.3.1. Friction Reduction Under Different Loads

**Figure 6:**
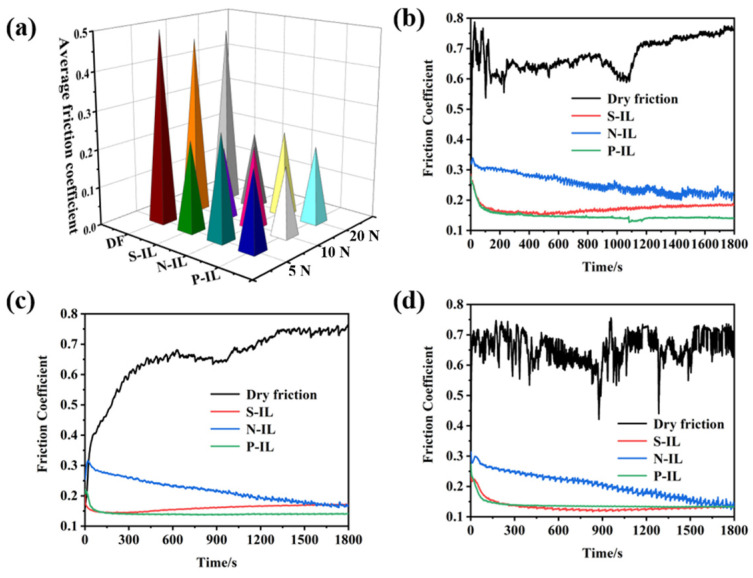
(**a**) Average friction coefficient of different ionic liquid lubricants under different loads, and different colors represent different samples under different loads; the friction coefficient curves with time dependent for different IL lubricants under 5 N (**b**), 10 N (**c**), and 20 N (**d**) load.

Ionic liquids generally form protective lubricating films at the friction interface, which alleviate sliding and thereby reduce friction and wear [39,40,41,42,43]. As shown in Figure 6, under different loading conditions, all three ionic liquids exhibited significant friction-reducing effects, with friction coefficients markedly lower than those observed under dry friction. However, their tribological behaviors varied due to differences in the polar groups within their molecular structures and the resulting interfacial interactions. P-IL, containing phosphate anions, demonstrated excellent friction-reducing performance across different contact stresses. This behavior can be attributed, on the one hand, to tribochemical reactions between the active elements (P and N) and the ceramic interface, which promote the formation of a boundary lubrication film. On the other hand, the relatively high hydroxyl content in its molecular structure facilitates hydrogen bonding, potentially generating a lubricating layer analogous to a hydrated film. For N-IL, the friction coefficient curve showed relatively high values and a prolonged running-in period. Notably, the friction coefficient gradually decreased with time. This trend may result from the absence of hydroxyl groups in its anionic structure, combined with the higher number of lone-pair electrons on oxygen, which increases the energy dissipation at the ceramic interface [44]. In the case of S-IL, the friction coefficient decreased initially and then increased at lower loads, while remaining stable after the running-in period at higher loads. This behavior may be related to the hydrolysis tendency of the sulfur-containing anion, which contributes to the instability of the friction coefficient in the later stages [45].

**Figure 7:**
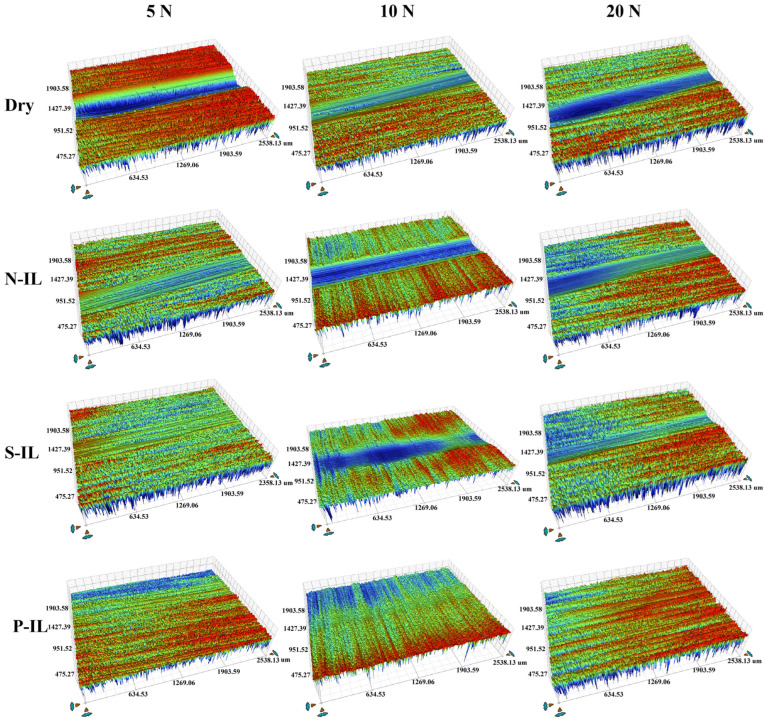
The three-dimensional topographies for the worn surface after lubrication by different IL lubricants under the load of 5 N, 10 N, and 20 N, respectively.

The wear of the ceramic surfaces lubricated by ionic liquids was characterized using three-dimensional profilometry, as shown in Figure 7. With an increasing load, the interfacial wear gradually intensified. Notably, P-IL exhibited excellent anti-wear performance under different contact stresses, with only minimal surface damage and no evident wear pits. This superior performance can be largely attributed to tribochemical reactions at the ionic liquid–ceramic interface, which facilitate the formation of boundary lubrication films that protect the surface [46].

#### 3.3.2. Friction-Reducing and Anti-Wear Properties on Different Friction Pairs

**Figure 8:**
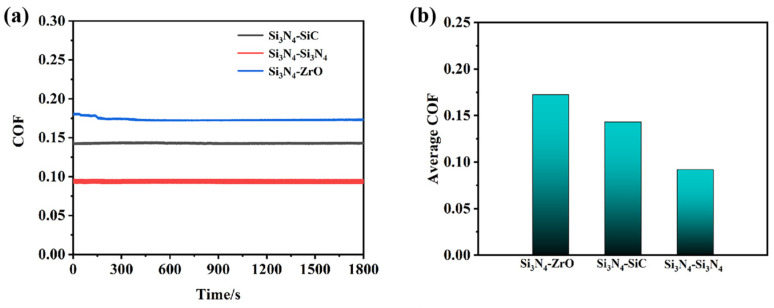
(**a**) Friction coefficient curves, and (**b**) average friction coefficients on Si_3_N_4_ substrates against ZrO_2_, SiC, and Si_3_N_4_, respectively.

**Figure 9:**
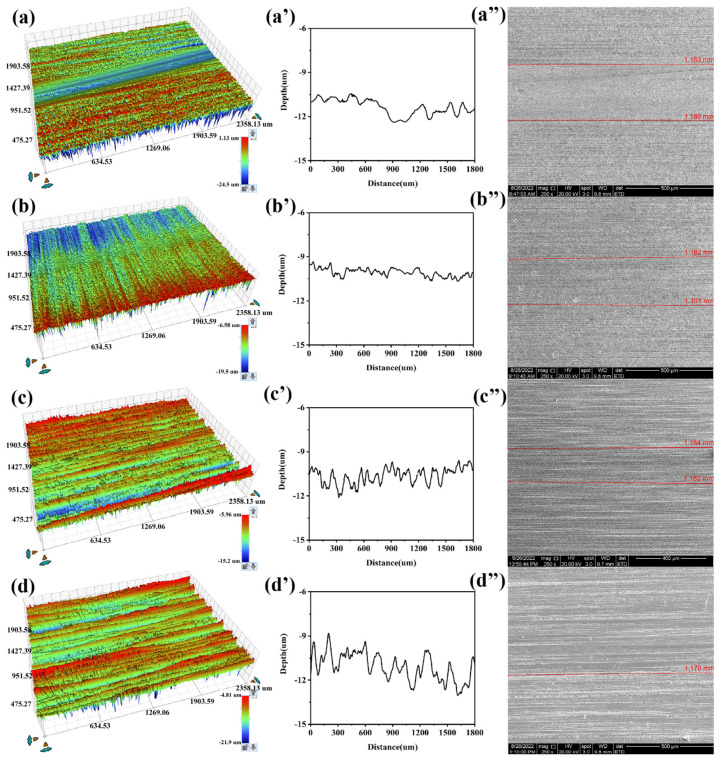
3D surface morphologies, cross-sectional profiles, and SEM images of wear tracks lubricated without IL (**a**,**a′**,**a″**) and with P-IL on SiC substrates against SiC (**b**,**b′**,**b″**), Si_3_N_4_ (**c**,**c′**,**c″**), and ZrO_2_ (**d**,**d′**,**d″**), respectively.

Ceramic materials inherently exhibit self-lubricating properties, and their lubrication mechanisms vary with the substrate type. Consequently, the co-lubrication behavior of ILs also differs across ceramic interfaces. Here, the tribological performance of P-IL, which demonstrated the best friction-reduction ability in previous tests, was further investigated on three ceramic substrates. As shown in Figure 8, the overall performance followed the trend: ZrO_2_ > Si_3_N_4_ > SiC. This trend arises not only from the tribochemical interactions between ILs and ceramic substrates but also from the intrinsic physicochemical properties of the substrates themselves [47]. Si_3_N_4_, with its combination of high hardness, toughness, and corrosion resistance, exhibited strong adaptability under harsh operating conditions (high loads and high temperature). In addition, its negatively charged surface provides abundant adsorption and reaction sites, facilitating the attachment of IL cations to the sliding interface. This process promotes the formation of an adsorbed electric double layer at the asperities during shear. As a result, more IL molecules can self-assemble on the surface, enhancing the load-bearing capacity and reducing the friction coefficient. Moreover, the hydroxyl-rich anionic moieties of P-IL promote the formation of a thicker protective film at the Si_3_N_4_ interface [48]. Accordingly, the wear scars on Si_3_N_4_ lubricated with P-IL were shallower than those on other substrates, demonstrating superior anti-wear performance. At the sliding interface, highly polar ILs also undergo tribochemical reactions, generating interfacial tribofilms containing nitrogen, oxygen, and phosphorus, as further confirmed by the subsequent XPS analysis. These tribofilms possess a bonding strength that prevents direct asperity–asperity contact, thereby reducing the surface damage [47]. As shown in Figure 9, the ZrO_2_ ceramic substrate exhibited more severe wear than the Si_3_N_4_ discs, which can be attributed to two main factors: te intrinsic physical properties and interfacial tribochemical reactions. Regarding the former, the lower load-bearing capacity of the ZrO_2_ matrix arises from its relatively low hardness, strength, and surface roughness. Consequently, the wear scars and surface damage on ZrO_2_ was deeper and wider than those on Si_3_N_4_ under the same IL lubrication. Concerning the later, the reactivity and interfacial interactions of different ceramics with ILs vary significantly. Si_3_N_4_ displayed higher molecular activity compared with ZrO_2_, which promoted interfacial tribochemical reactions. This facilitated the formation of adsorbed electric double layers and tribochemical lubricating films at the sliding asperities, thereby enhancing its wear resistance [49].

### 3.4. Surface Analysis (SEM)

**Figure 10:**
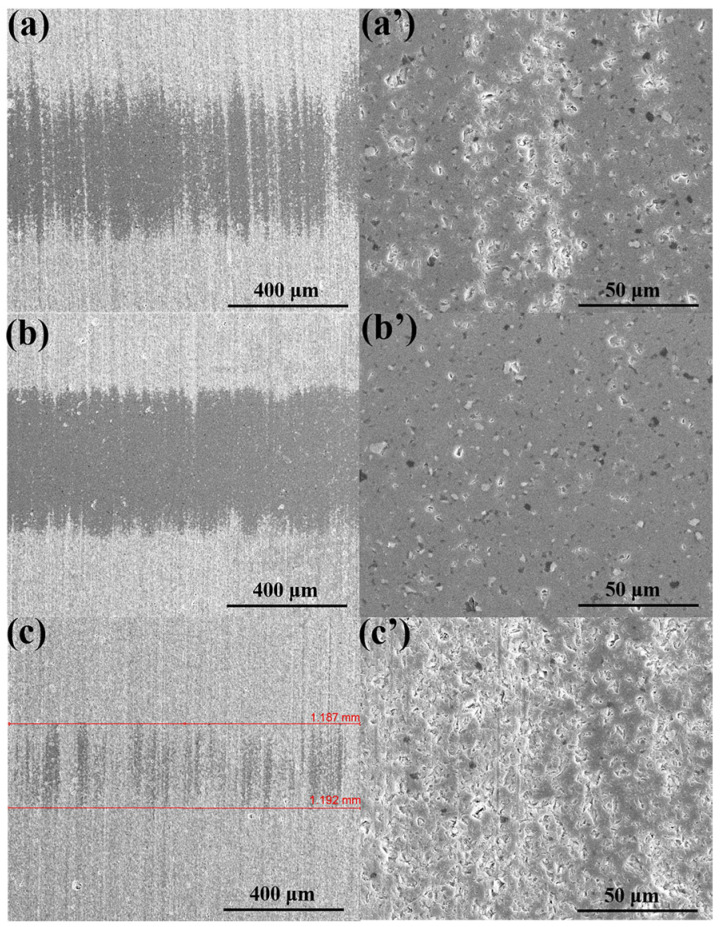
The SEM picture of wear tracks for P-IL on Si_3_N_4_ substrates against (**a**,**a′**) ZrO_2_, (**b**,**b′**) SiC, and (**c**,**c′**) Si_3_N_4_, respectively.

Figure 10 presents the SEM images of the worn surfaces of SiC discs lubricated with different ILs, revealing distinct surface morphologies. For the Si_3_N_4_ sliding interface lubricated with P-IL, the wear scars were smooth and well-preserved. The faint scratches observed were mainly due to the initial polishing treatment of the ceramic discs rather than chemical attack, indicating that P-IL effectively protected the interface. In contrast, slight sliding scars were visible at the SiC interface lubricated with P-IL, while the worn Si_3_N_4_ surfaces appeared rougher and showed signs of deterioration, with localized adhesive wear on the substrate. These observations were consistent with the 3D optical profilometry results. As the friction progressed, the wear mechanism at the Si_3_N_4_ interface transitioned from being mechanically dominated to tribochemically dominated [50]. Overall, P-IL exhibited excellent anti-wear properties and load-bearing capacity. The enhanced corrosion- and wear-resistant performance on SiC substrates is attributed to their intrinsic properties, including the high hardness, strong reactivity, and corrosion resistance, which enable them to maintain stability under high-load and high-temperature conditions. After sliding, the negatively charged Si_3_N_4_ surfaces provided abundant active sites for the adsorption of IL molecules. This promoted the formation of an electric double layer, which played a key role in improving wear resistance and lubrication stability. Moreover, a large number of IL molecules became enriched and adsorbed onto the protective layer, increasing molecular coverage and enhancing the film stability and bonding strength. In addition, the bulkier anionic moieties of P-IL, compared with those of S-IL, contributed to stronger interfacial interactions and adsorption stability, ultimately leading to the formation of a thicker and more stable tribofilm at the sliding asperities.

### 3.5. Wetting Performance

**Figure 11:**
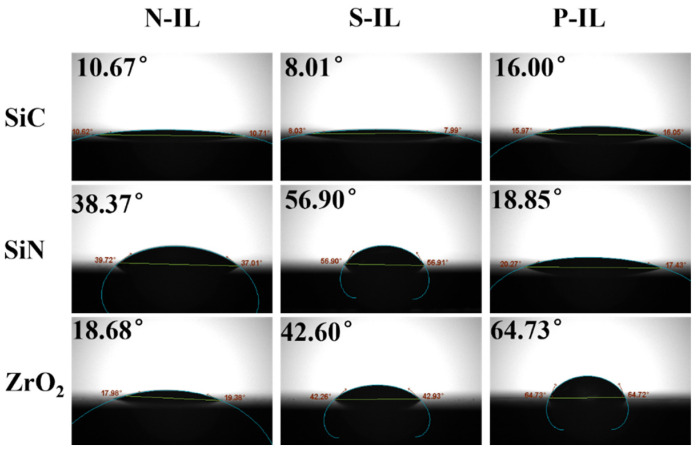
Contact angle values and photographs of ionic liquids on different ceramic substrates.

Wettability is also a key factor for the formation of efficient and stable adsorption films at the contact interface. The contact angle measured on the ceramic surface is a critical parameter for evaluating the wettability of ionic liquid lubricants. A smaller contact angle corresponds to better wettability, facilitating spreading at the contact interface and promoting film formation [51]. As shown in Figure 11, the contact angles of the ILs on ceramic surfaces were less than 90°, indicating that the surfaces were generally hydrophilic and that the lubricants could wet the substrates effectively. However, the magnitude of the contact angles varied among the different ceramics. On the SiC surface, all three ionic liquids exhibited very small contact angles, reflecting the superior spreading ability and confirming the inherent hydrophilicity of SiC. For Si_3_N_4_, the order of contact angle values was P-IL > N-IL > S-IL, consistent with the observed tribological performance. Tribological systems with smaller contact angles typically demonstrate improved lubrication, since enhanced wettability accelerates the spreading and adsorption of ILs within the real contact area. By contrast, the larger contact angles measured on ZrO_2_ surfaces suggested weaker wetting behavior, which may account for its relatively poor tribological performance compared with SiC and Si_3_N_4_.

### 3.6. XPS Analysis

#### 3.6.1. Si_3_N_4_ Ceramic

**Figure 12:**
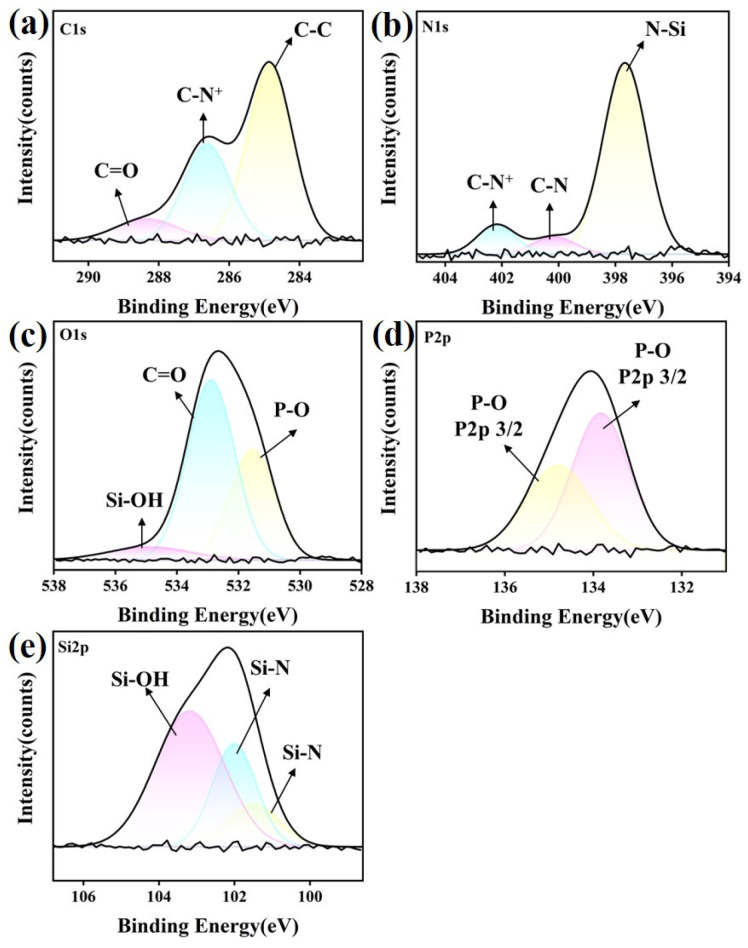
XPS spectra concerning (**a**) C 1s, (**b**) N 1s, (**c**) O 1s, (**d**) P 2p, and (**e**) Si 2p of Si_3_N_4_ ceramic substrate after lubrication by P-IL.

The XPS spectra of the characteristic elements (C 1s, N 1s, P 2p, O 1s, and Si 2p) were analyzed to investigate the lubricating mechanism and interfacial interaction of IL lubricants for the Si_3_N_4_ substrate. The analysis was carried out on the worn surfaces lubricated with P-IL after friction tests. As shown in Figure 12, the carbon spectrum could be deconvoluted into three peaks at 285.7 eV, 284.2 eV, and 282.5 eV, corresponding to C=O, C–N^+^, and C–C bonding, respectively. Meanwhile, a distinct P 2p peak appeared at 131.6 eV after the friction test. In addition, the O 1s spectrum exhibited peaks at 531.3 eV (Si–OH), 530.6 eV (C=O), and 529.6 eV (P–O), indicating the formation of phosphorus oxides and PO_4_^3^^−^ species at the sliding asperities. The N 1s spectrum showed binding energies at 399.8 eV (C–N^+^), 397.8 eV (C–N), and 395.4 eV (N-Si), further confirming the interfacial tribochemical reactions between P-IL and the Si_3_N_4_ substrate during shear [52]. For the Si 2p spectrum, the peaks at 101.8 eV (Si–OH) and 100.7 eV (Si–O–P) were more intense than that of Si–C, suggesting that extensive tribochemical reactions occurred. The presence of Si–OH implies the possible formation of a hydrated layer-like structure, which could further enhance lubrication [53]. Under the applied test conditions, the oxidation kinetics of Si_3_N_4_ played a positive role, explaining why the lubricity of P-IL on the Si_3_N_4_ contacts was superior to that on the SiC counterparts. During friction and shear, the higher electron and defect emission from the Si_3_N_4_ substrate promoted faster oxidation compared with SiC under the same conditions [54]. The formation of silicate on the Si_3_N_4_ substrate facilitated the generation of thin films and silicate clusters on the interface, improving the viscosity and lubrication performance. Therefore, the excellent tribological performance of P-IL can be attributed to interfacial tribochemical reactions on the Si_3_N_4_ substrate, further supported by the formation of an electric double layer [55].

#### 3.6.2. ZrO_2_ Ceramic

**Figure 13:**
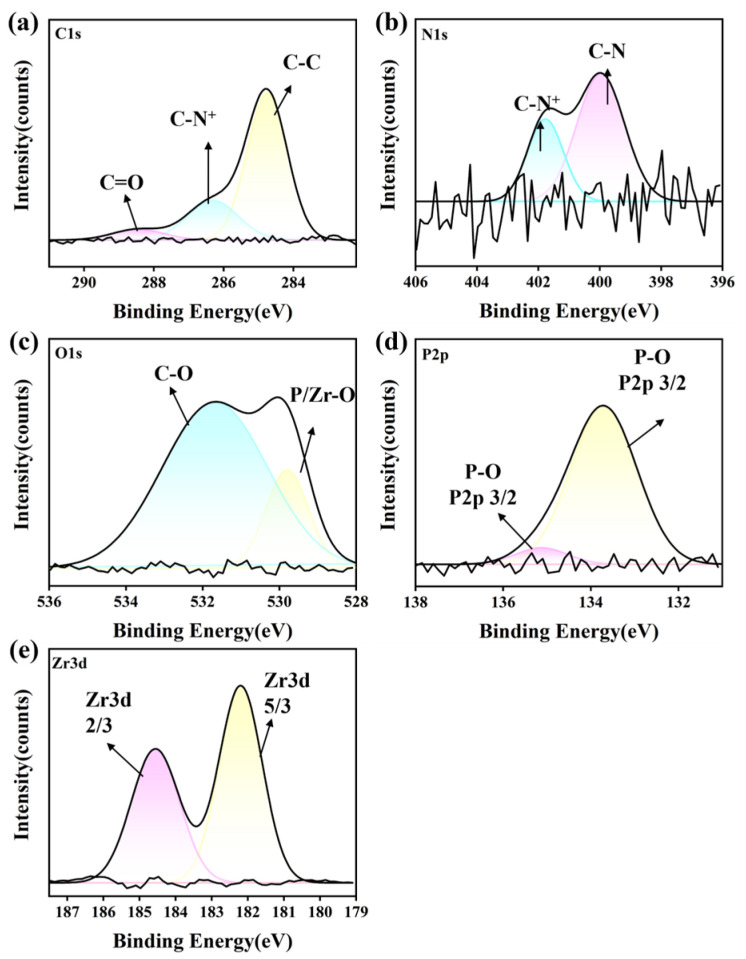
XPS spectra concerning (**a**) C 1s, (**b**) N 1s, (**c**) O 1s, (**d**) P 2p, and (**e**) Zr 3d of ZrO_2_ ceramic substrate after lubrication by P-IL.

For the Si_3_N_4_–ZrO_2_ contact pair, an XPS analysis was conducted to further clarify the tribological mechanism, and the corresponding spectra are shown in Figure 13. The typical elements detected included C 1s, N 1s, P 2p, O 1s, and Zr 3d on the ZrO_2_ surfaces after lubrication with P-IL. The C 1s spectrum exhibited peaks at 286.2 eV (C=O), 284.3 eV (C–N^+^), and 282.6 eV (C–C). The N 1s spectrum showed peaks at 399.7 eV (C–N^+^) and 397.9 eV (C–N), which can be attributed to the cationic portion, indicating changes in the nitrogen chemical state during friction. This suggests that the ionic liquid decomposed under sliding conditions and actively participated in tribochemical reactions with the ZrO_2_ substrate. For O 1s, the main peaks were assigned to C=O (530.7 eV), P–O (528.9 eV), and Zr–O (527.8 eV) bonding. In addition, a clear P 2p peak was observed at 131.7 eV, corresponding to phosphorus oxides such as Zr(HPO_4_)_2_ and ZrP_2_O_7_, as well as complex compounds containing Zr, O, C, P, and H. At a given pH, the negative surface charge density of ZrO_2_ increases with rising temperature, which enhances the electrostatic repulsion with the anionic groups of P-IL. As a result, a compact electric double layer cannot form. However, the interaction with cationic moieties is strengthened, leading to a higher proportion of C–N^+^ fragments. Friction further promotes C=O bond cleavage, generating additional C–N fragments [56]. Taken together, these results demonstrate that P-IL actively reacts with ZrO_2_-based contact surfaces during sliding, leading to the formation of a protective tribofilm that contributes to improved tribological performance.

#### 3.6.3. SiC Ceramic

**Figure 14:**
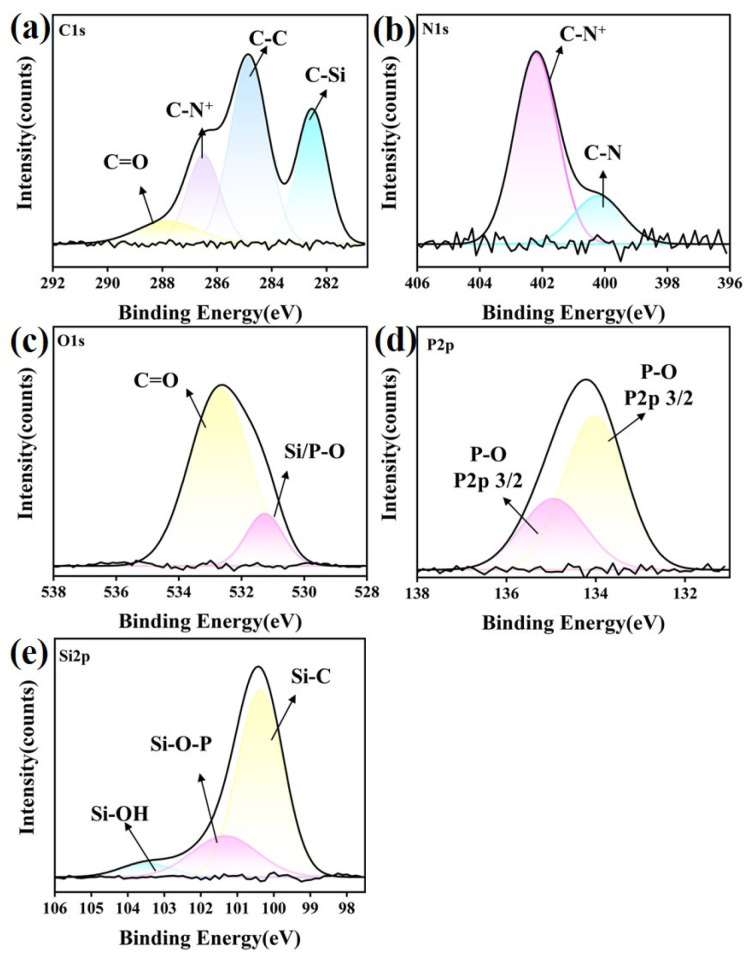
XPS spectra concerning (**a**) C 1s, (**b**) N 1s, (**c**) O 1s, (**d**) P 2p, and (**e**) Si 2p of SiC ceramic substrate after lubrication by P-IL.

As shown in Figure 14, the front edge of the carbon spectrum on the SiC substrate exhibited distinct peaks located at 287.4 eV (C=O), 286.2 eV (C–N^+^), 284.7 eV (C–C), and 282.5 eV (C–Si). The formation of C–N^+^ was mainly attributed to the decarbonylation of aldehyde groups in the cationic moieties under friction, as well as the chemical bonding of amine groups to the counterface or the formation of surface polymers. A large amount of phosphate was also detected at the interface, as evidenced by the phosphorus spectrum. In the Si 2p region, peaks at 103.2 eV (Si–OH) and 101.4 eV (Si-O-P) indicated that tribochemical reactions occurred at the frictional interface during sliding. Therefore, P-IL lubricates the SiC substrate primarily through the formation of an adsorbed electric double layer.

### 3.7. Lubrication Mechanism

**Figure 15:**
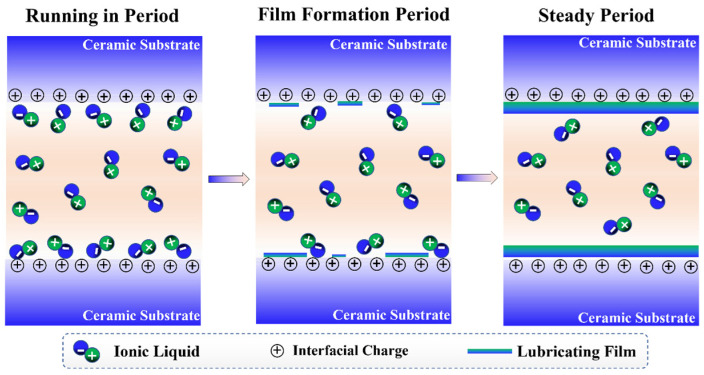
Schematic diagram of lubrication mechanism.

P-IL exhibited superior tribological properties compared with N-IL and S-IL in all tested ceramic contacts (Si_3_N_4_–SiC_,_ Si_3_N_4_–Si_3_N_4_, and Si_3_N_4_–ZrO_2_). Its excellent performance can be attributed to the self-assembled boundary protective films formed at the sliding asperities, consisting of both interfacial tribochemical reactions and electric double-layer structures, as shown in Figure 15. The film thickness is closely related to the contact geometry, sliding velocity, and lubricant viscosity. According to the tribological characterization and the preceding analyses, highly polar IL molecules exhibit a strong adsorption and high surface coverage on ceramic interfaces. Under the combined effects of frictional heat and mechanical shear, interfacial tribochemical reactions occur, leading to the formation of boundary films with a low shear strength and high load-bearing capacity. Thus, boundary lubrication is the dominant lubrication mode under IL lubrication [39]. The superior performance of P-IL results from multiple interactions. First, physical transfer occurs, in which small debris particles or IL molecules are mechanically deposited onto the ceramic surface during sliding. Second, chemical deposition becomes increasingly significant with prolonged friction, as active functional groups in the ILs react with ceramic surfaces to generate new adherent compounds. Third, polar adsorption enables IL molecules to rapidly attach to the ceramic interface, enhancing coverage during the running-in period. Under continued shear, these processes cooperatively promote the gradual formation of stable boundary tribofilms. Mechanical wear simultaneously disturbs the films, accelerating tribochemical reactions that further reinforce the protective layer. Ultimately, a sufficiently thick tribofilm is established, effectively preventing direct asperity contact between friction pairs. Importantly, the thickness and viscoelasticity of the adsorbed layer are positively correlated with the molecular structures of the ILs. Although the synthesized ILs share the same cation, the larger volume and stronger adsorption capability of the phosphate anion in P-IL allow it to form a more robust and stable protective layer compared with S-IL. This structural advantage, coupled with the high viscosity of P-IL, contributes to its enhanced lubricating stability, superior anti-wear performance, and improved load-carrying capacity in practical applications.

## 4. Conclusions

Three novel ionic liquids were designed and synthesized as efficient lubricants for ceramic friction couples, and their physicochemical and tribological properties were systematically investigated. The study primarily focused on the tribological behavior of P-IL on three ceramic contacts: SiC, Si_3_N_4_, and ZrO_2_. The results demonstrated that P-IL exhibited the best performance on Si_3_N_4_–Si_3_N_4_ contacts, achieving superior friction reduction with an over 80% decrease in friction coefficient and wear resistance with a reduced wear volume compared with Si_3_N_4_–ZrO_2_ and Si_3_N_4_–SiC contacts. This advantage was attributed to the higher hardness, favorable intrinsic structure, and stronger surface activity of Si_3_N_4_. The excellent physicochemical properties of the synthesized ILs provided a strong foundation for their tribological performance. XPS, SEM, and CA analyses confirmed that the tribological behaviors of the ILs were closely related to their molecular structure and interface interactions. Among them, the highly polar P-IL exhibited stronger adsorption and interfacial interactions, resulting in improved lubrication and load-bearing capacity compared with N-IL and S-IL across all tested ceramic contacts. Mechanistically, the as-synthesized ILs primarily functioned through interfacial adsorption and tribochemical reactions. These processes promoted the formation of boundary lubrication films, which effectively reduced friction, enhanced wear resistance, and improved load-bearing performance under shear.

## Figures and Tables

**Table 1:** Thermal decomposition temperature curve of ionic liquid lubricants.

Samples	Viscosity (mm^2^/s), 25 °C
N-IL	2.43
S-IL	7.98
P-IL	95.75

## Data Availability

The original contributions presented in this study are included in the article. Further inquiries can be directed to the corresponding authors.
